# Correlation between gene polymorphism and adverse reactions of high-dose methotrexate in osteosarcoma patients: a systematic review and meta-analysis

**DOI:** 10.1186/s12957-023-03287-0

**Published:** 2024-01-11

**Authors:** Ben Liu, Gang Liu, Binbin Liu, Yao Guo, Ningning Peng, Tiejun Li

**Affiliations:** 1 Fourth Department of Orthopedics, Cangzhou Central Hospital, No. 16 Xinhua West Road, Canal District, Cangzhou, HeBei 061000 China; 2 Central Laboratory, Cangzhou Central Hospital, Cangzhou, HeBei China; 3 Legal Affairs Office, Cangzhou Central Hospital, Cangzhou, HeBei China

**Keywords:** High-dose Methotrexate, Adverse Reactions, Osteosarcoma, Gene Polymorphism, Meta-analysis

## Abstract

**Objective:**

We aimed to provide a reference based on evidence for an individualized clinical medication of high-dose methotrexate (HD-MTX) in osteosarcoma patients by evaluating the effect of gene polymorphism on adverse reactions of HD-MTX usage.

**Methods:**

Several databases were combed for research on the association between gene polymorphisms and adverse reactions to HD-MTX up to January 2023. A meta-analysis and/or descriptive analysis on the incidence of HD-MTX-related adverse reactions were conducted by using clinical studies meeting inclusion criteria.

**Results:**

Twelve studies involving 889 patients were included. There were 8, 6, 5, and 4 studies related to MTHFR C677T, MTHFR A1298C, RFC1 G80A, and MDR1 C3435T polymorphisms, respectively. The results of the meta-analysis showed that the MTHFR C677T polymorphism was associated with G3-4 hepatotoxicity, G3-4 nephrotoxicity, G3-4 gastrointestinal toxicity, and G3-4 mucositis under the recessive genetic model (MM vs. Mm/mm). Limited research showed that MTHFR C677T was associated with G3-4 nephrotoxicity in the allelic genetic model (M vs. m). MTHFR A1298C polymorphism was associated with a decreased risk of adverse reactions to HD-MTX usage, without statistical significance. This review's descriptive analysis showed no significant correlation between the RFC1 G80A, and MDR1 C3435T polymorphism and adverse reactions of HD-MTX.

**Conclusion:**

The MTHFR C677T mutation may enhance the risk of HD-MTX adverse reactions in osteosarcoma patients. Existing studies have not found a significant correlation between the MTHFR A1298C, RFC1 G80A, and MDR1 C3435T polymorphism and adverse reactions caused by HD-MTX. Lastly, this conclusion was limited because of few studies.

**Supplementary Information:**

The online version contains supplementary material available at 10.1186/s12957-023-03287-0.

## Introduction

Osteosarcoma is the most frequent malignant primary bone tumor in children and adolescents. The clinical treatment for osteosarcoma is still a challenge which consists primarily of surgical treatment, complemented by chemotherapy or other treatments [1]. For the chemotherapy regimen of osteosarcoma, the National Comprehensive Cancer Network’s “Clinical Practice Guidelines for Bone Tumors,” the European Society for Medical Oncology’s “Guidelines for Diagnosis, Treatment, and Follow-up of Osteosarcoma,” and the “Chinese Clinical Evidence-based Diagnosis and Treatment Guidelines for Osteosarcoma (2018)” all recommend HD-MTX intravenous injection as the first-line chemotherapy drug, that is, a dose > 500 mg/m^2^ [2–5].

Postoperative administration of HD-MTX alone or combined with other chemotherapeutic agents can increase the survival rate of osteosarcoma patients[6]. Extracellular metabolites of HD-MTX include 7-hydroxymethotrexate and 2,4-diamino-N10 methylbutyrate. Intracellular metabolites of HD-MTX include polyglutamate methotrexate [7, 8]. In osteosarcoma chemotherapy, HD-MTX can effectively increase blood concentration and enhance the curative effect of osteosarcoma chemotherapy while the incidence of adverse drug reactions increases proportionally. These extreme adverse reactions include hematotoxicity, hepatotoxicity, and nephrotoxicity. The pharmacokinetics of MTX vary considerably between patients. Therefore, individual variations in efficacy and adverse reactions can result in chemotherapy interruption and tumor recurrence [9].

Research has shown that pharmacogenomics significantly influences the pharmacokinetics of Methotrexate (MTX), leading to variations in patient responses and side effects. This variation is largely due to gene polymorphisms in MTX metabolic enzymes, transporters, and target proteins [10]. Key metabolic enzymes involved in MTX processing include methylenetetrahydrofolate reductase (MTHFR), lutein acyl polyglutamate synthase, γ-glutamyl hydrolase, and the cytochrome P450 system. The main transporters associated with MTX are the adenosine triphosphate-binding cassette transporter, solute carrier 19A1, and organic anion transporting polypeptide 1B1, while its primary targets are dihydrofolate reductase and thymidylate synthase [11–13]. Significant research has focused on gene polymorphisms related to MTHFR, reduced folate carrier (RFC), and P-glycoprotein in the MTX metabolic pathway. MTHFR, crucial in intracellular folate metabolism, affects MTX's pharmacological impact, with common polymorphisms being MTHFR C677T and A1298C (rs1801133/ rs1801131) [11]. The RFC1/ SLC19A1 gene, vital for MTX cellular transport, exhibits polymorphism in RFC1 G80A (rs1051266) [12]. P-glycoprotein (P-gp), encoded by the multidrug resistance gene (MDR1/ ABCB1), plays a role in MTX efflux from cells, with MDR1 C3435T (rs1045642) as a notable polymorphism [13]. These genetic variations significantly affect MTX pharmacokinetics and patient responses.

Currently, the correlation between gene polymorphisms related to MTX metabolism and pharmacological effects and HD-MTX adverse reactions is inconsistently supported by research. For instance, the MTHFR 677 CT/ TT genotype in the Chinese population substantially increased the risk of moderate to severe oral mucositis [14]. In contrast, no significant correlation was observed in the Belgian population [15]. In patients with osteosarcoma, there still needs to be more practical guidance regarding HD-MTX individualized medication. However, there is no systematic evaluation of the effect of related gene polymorphisms on HD-MTX adverse reactions in patients with osteosarcoma. The purpose of this meta-analysis was to comprehensively evaluate the effects of MTX metabolism and pharmacological effects related to gene polymorphisms on HD-MTX adverse reactions and to provide an evidence-based reference for the clinical use of HD-MTX in osteosarcoma patients. It can provide an evidence-based basis for therapeutic choices, promote individualized HD-MTX treatment, and ultimately enhance the clinical outcome of osteosarcoma patients.

## Methods

The protocol of this meta-analysis was registered on the International Prospective Register of Systematic Reviews (PROSPERO ID: CRD42023444896). We reported it by the PRISMA-P (Preferred Reporting Items for Systematic Reviews and Meta-Analyses Protocols) statement (supplemental material 1) [16].

### Inclusion criteria

Published prospective and retrospective studies were included in this study. The subjects were osteosarcoma patients receiving HD-MTX chemotherapy, irrespective of race, gender, or age. The exposure factors were gene polymorphisms associated with MTX metabolism and pharmacological effects, and osteosarcoma patients were categorized according to the wild-type and mutant-type. The outcome indicator was the incidence of HD-MTX-related adverse reactions, including Hematotoxicity (the incidence of leukopenia, granulocytopenia, thrombocytopenia and hemoglobin reduction/anemia), hepatotoxicity, nephrotoxicity, mucositis, gastrointestinal toxicity, and overall adverse events. According to the fifth edition of the World Health Organization (WHO) anticancer drug toxicity grading, the US National Cancer Institute's standard adverse reaction evaluation criteria and common terminology criteria for adverse events (CTCAE), adverse reactions were graded as G1-2 (mild and moderate) and G3-4 (severe).

### Exclusion criteria

This research excluded duplicate publications or literature with repeated samples. After contacting the author, studies that could not obtain the full text or correct data would also be excluded. This study did not include studies whose outcome indicators were limited to overall survival and the risk of recurrence of the primary disease. Studies that did not provide a genotype or a genotype distribution that meets the Hardy–Weinberg equilibrium would be excluded from the meta-analysis [17].

### Retrieval strategy

Databases including Pubmed, Web of Science, Cochrane, CNKI, and Wanfang Data were searched (Accomplished by Liu B and Liu G). Database retrieval was limited to topics, abstracts, and keywords. The terms were ‘Methotrexate,’ ‘Osteosarcoma,’ ‘Gene,’ ‘Polymorphism,’ ‘Methylenetetrahydrofolate reductase,’ ‘RFC1,’ ‘P-glycoprotein,’ ‘Multidrug resistance protein 1.’ The details of the search strategy were provided in supplemental material 2. The retrieval time limit is from establishing the database until January 2023. At the same time, the researchers (Liu B, Liu BB and Guo Y) also manually searched the references included in the literature.

### Data extraction

After clarifying and integrating the inclusion and exclusion criteria for the literature, two researchers (Peng NN and Li TJ) independently screened the literature by title, abstract, and full text. After concluding the literature screening, the two researchers independently extracted the fundamental data from the included literature using the pre-designed data extraction table. The extracted data included the first author, publication year, patient's country, race, or region, gender, age, MTX dose, gene locus, conformance to Hardy–Weinberg equilibrium (chi-square test), outcome indicators, and definition of adverse effects. If pertinent data was lacking from the included literature, contact the corresponding author via email to obtain the data. When data extraction was inconsistent, the issue was resolved through discussion or consultation with a third researcher (Liu G).

### Quality assessment

Two researchers (Peng NN and Li TJ) independently applied the Cochrane Collaboration-recommended Newcastle–Ottawa Scale (NOS) to assess the risk of bias in the included studies [18]. The NOS scale consisted of 8 evaluation items with a total score of 9. There were 4 items to the study population selection, 1 to the comparability between groups, and 3 to the outcome measurement. Studies with scores 7 to 9 were of high quality, 5 to 6 were of medium quality, and 1 to 4 were of low quality. If there were any objections, a third researcher (Liu G) engaged in the discussion and resolved the differences.

### Statistical method

We utilized Stata 15.1 software to process the literature data [19, 20]. The χ2 test was used to determine the genotype distribution frequency of the included literature. *P* > 0.05 indicated that the sample was in Hardy–Weinberg equilibrium, suggesting that the sample was representative of the group. First, it was determined whether there was apparent clinical heterogeneity between the studies of the same outcome index, including whether the categories of diseases and definitions of adverse reactions were comparable. We performed a meta-analysis on the outcome index when two or more studies reported the same outcome, and there was no significant clinical heterogeneity between studies. Descriptive analysis was conducted for outcome indicators that were not amenable to meta-analysis. The odds ratio (OR) and its 95% confidence interval (CI) were applied to assess the relationship between gene polymorphism and HD-MTX adverse reactions. In addition, we would calculate the OR and 95% CI under the dominant genetic model (MM/Mm vs. mm), recessive genetic model (MM vs. Mm/mm), and allele genetic model (M vs. m), where M is the mutant type and m is the wild type. In this study, the Q test and *I*^*2*^ test were used to examine the heterogeneity of the research results included [21]. If *I*^*2*^ > 50%, the heterogeneity of the included studies was low. In contrast, *I*^*2*^ ≥ 50% indicated that the included studies were highly heterogeneous. If *I*^*2*^ < 50%, the fixed effect model was used for the meta-analysis, and the random effect model was applied if *I*^*2*^ ≥ 50%. In the present study, subgroup analysis was conducted according to race to determine whether there were statistically significant differences between subgroups. To test publication bias, we utilized Egger's linear regression [22]. Duval and Tweedie's trim and fill test evaluated the results' sensitivity. Suppose the combined effect size was spliced before and after the test, and the combined effect size significantly changed. In that case, the research results were unreliable, and additional analysis of the merged research is required [23]. Bilateral *P* < 0.05 was considered statistically significant in this study. Unless *P* < 0.001, we would provide an exact value for *P*.

## Results

### Literature screening, basic characteristics and quality assessment

**Fig. 1:**
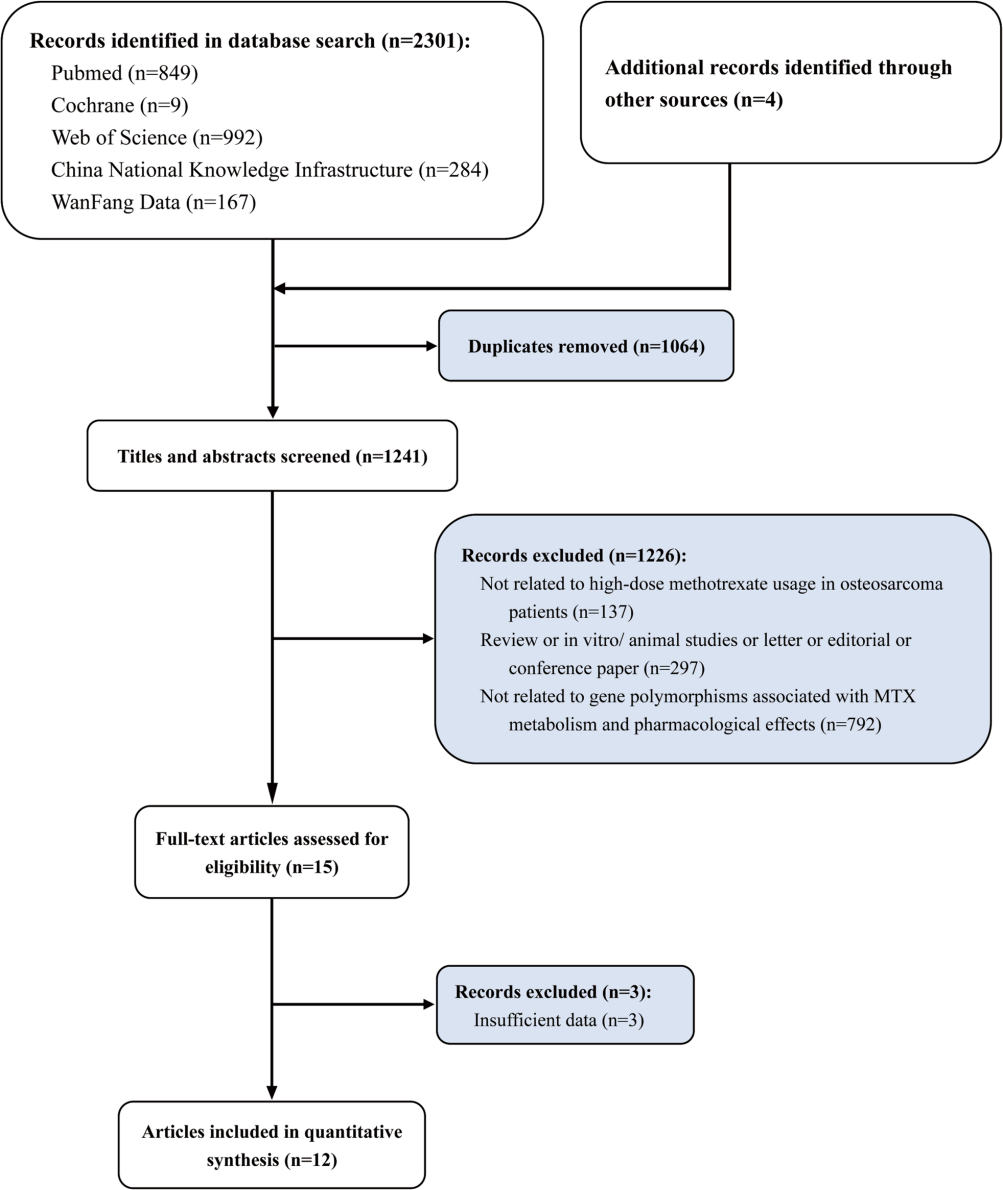
Study selection flowchart

According to the respective retrieval strategies, 2301 studies were retrieved, and 4 were added manually. 12 studies were ultimately included after 1064 repeated studies were eliminated and 1229 studies were eliminated after reading titles and abstracts. Basic characteristics and reasons for excluded three studies in the full-text assessment were provided in supplemental Table 1. With a total of 985 patients, 8 studies were included in the meta-analysis and 4 studies were only included in the descriptive analysis (Fig. 1). 6 of the 12 included studies were concerned with Asian populations, 7 with Caucasian people. 12 studies were exclusively limited to HD-MTX in terms of dosage. There were 8, 6, 5, and 4 studies on MTHFR C677T, MTHFR A1298C, RFC1 G80A, and MDR1 C3435T regarding gene polymorphism, respectively. Following the χ^2^ test, 12 included studies were consistent with HWE. Hematological toxicity, hepatotoxicity, nephrotoxicity, gastrointestinal toxicity, mucositis, and overall adverse reactions were reported as outcome indicators. The classification criteria for adverse reactions consisted primarily of WHO or CTCAE criteria. Table 1 shows basic information about the included studies [15, 24–34].

**Table 1:** Baseline characteristics of included studies for meta-analysis

First author, year	Country	Race	Sample size	Sex		Gene polymorphism	Outcome indicators	Classification criteria	NOS score
				**Male**	**Female**				
Hattinger CM, 2016 [24]	Italy	Caucasian	57	37	20	α, β	ε, ι, ζ, η	CTCAE	8
Ren HY, 2011 [14, 25]	China	Asian	210	109	101	γ	ε, θ, ι, η	WHO	8
Windsor RE, 2012 [26]	United Kingdom	Caucasian	58	34	24	α, β	ε, θ	CTCAE	7
Jabeen S, 2015 [27]	Norway	Caucasian	62	-	-	γ, α, δ	θ, ζ	CTCAE	7
Park JA, 2016 [28]	Korea	Asian	37	21	17	γ, α, δ	θ, ζ, η	CTCAE	6
Goricar K, 2014 [29]	Slovenia	Caucasian	74	38	36	β, δ	ε, ζ	CTCAE	6
Xu L, 2018 [30]	China	Asian	109	58	51	γ	θ, ζ, η, ε	CTCAE	8
Lambrecht L, 2017 [15]	Belgium	Caucasian	48	23	25	γ	θ, ζ, η, ε	CTCAE	6
Wei Y, 2022 [31]	China	Asian	39	24	15	γ	θ, ζ, η, ι	CTCAE	9
Zhou XK, 2013 [32]	China	Asian	40	23	17	γ, α	θ, ζ, η, ι, ε	CTCAE	7
Hegyi M, 2017 [33]	Hungarian	Caucasian	59	-	-	δ, β	ε, ζ	CTCAE	7
Patino-Garcia A, 2009 [34]	Spain	Caucasian	96	41	55	γ, α, δ	ε, η, ι	WHO	9

α MTHFR A1298C, β MDR1 C3435T; γ MTHFR C677T; δ RFC1 G80A, ε Hematotoxicity, ζ Hepatotoxicity, η Nephrotoxicity, θ Mucositis, ι Gastrointestinal toxicity, *CTCAE* Common terminology criteria for adverse events, *WHO* World Health Organization

The NOS scores of the 12 included studies ranged from 6 to 9 points, indicating that they were all of moderate to high quality. 2 studies scored 9 points, 3 scored 8 points, 4 scored 7 points, and 3 scored 6 points. Three studies did not characterize follow-up and loss of follow-up [15, 28, 29]. All studies meticulously considered the selection of population and comparability between cohorts. Table 1 displays the outcomes of methodological quality evaluations of the included studies [15, 24–34]. The details of the NOS score assessment were provided in supplemental Table 2.

**Table 2:** Meta-analysis results of Correlation between MTHFR C677T Polymorphism and Adverse Reactions of HD-MTX

Indicators	CT/TT vs. CC					TT vs. CT/CC					T vs. C				
	No. of studies	No. of patients	OR (95%CI)	Heterogeneity		No. of studies	No. of patients	OR (95%CI)	Heterogeneity		No. of studies	No. of patients	OR (95%CI)	Heterogeneity	
				P	*I*^*2*^				P	*I*^*2*^				P	*I*^*2*^
G3-4 Hepatotoxicity	2	97	0.53 (0.18, 1.57)	0.230	30.5%	3	196	1.88 (1.39, 2.56)	0.772	0.0%	2	97	0.64 (0.15, 2.64)	0.078	67.9%
Asian	1	35	1.13 (0.22, 5.67)	-	-	2	148	1.94 (1.41, 2.67)	0.928	0.0%	1	35	1.32 (0.42, 4.15)	-	-
Caucasian	1	62	0.35 (0.13, 0.99)	-	-	1	48	1.23 (0.36, 4.15)	-	-	1	62	0.31 (0.10, 0.96)	-	-
G3-4 Nephrotoxicity	2	247	1.82 (0.70, 4.75)	0.008	85.9%	4	406	1.72 (1.10, 2.67)	0.096	52.8%	1	210	2.26 (1.25, 4.08)	-	-
Asian	2	247	1.82 (0.70, 4.75)	0.008	85.9%	3	358	1.79 (1.00, 3.22)	0.042	68.4%	1	210	2.26 (1.25, 4.08)	-	-
Caucasian	-	-	-	-	-	1	48	1.54 (0.69, 3.43)	-	-	-	-	-	-	-
G3-4 Gastrointestinal toxicity	1	210	3.43 (0.88, 13.32)	-	-	2	249	1.85 (1.34, 2.56)	0.319	0.0%	1	273	2.25 (0.79, 6.40)	-	-
Asian	1	210	3.43 (0.88, 13.32)	-	-	2	249	1.85 (1.34, 2.56)	0.319	0.0%	1	273	2.25 (0.79, 6.40)	-	-
Caucasian	-	-	-	-	-	-	-	-	-	-	-	-	-	-	-
G3-4 Mucositis	2	247	1.62 (0.73, 3.60)	0.057	72.5%	4	406	1.73 (1.31, 2.28)	0.839	0.0%	1	273	1.85 (0.92, 3.69)	-	-
Asian	2	247	1.62 (0.73, 3.60)	0.057	72.5%	3	358	1.77 (1.33, 2.35)	0.751	0.0%	1	273	1.85 (0.92, 3.69)	-	-
Caucasian	-	-	-	-	-	1	48	1.33 (0.47, 3.74)	-	-	-	-	-	-	-
G3-4 Hematotoxicity	1	220	2.67 (0.67, 10.59)	-	-	3	367	1.67 (0.95, 2.92)	0.335	8.7%	1	283	2.05 (0.67, 6.27)	-	-
Asian	1	220	2.67 (0.67, 10.59)	-	-	2	319	2.04 (0.70, 5.88)	0.148	52.2%	1	283	2.05 (0.67, 6.27)	-	-
Caucasian	-	-	-	-	-	1	48	1.45 (0.57, 3.71)	-	-	-	-	-	-	-

### Correlation between MTHFR C677T polymorphism and adverse reactions of HD-MTX

A total of 8 studies analyzed the association between MTHFR C677T gene polymorphism and HD-MTX adverse effects. The meta-analysis results for hepatotoxicity, nephrotoxicity, hematotoxicity, gastrointestinal toxicity, and mucositis are presented in Table 2.

For the incidence of G3-4 hepatotoxicity, only a meta-analysis of Asians under the recessive genetic model suggested that significant association between the MTHFR C677T polymorphism and hepatotoxicity (TT vs. CT/CC: OR = 1.94, 95%CI: 1.41–2.67) (Table 2). The results of the rest of the genetic model were non-significant differences, no matter in Asians or Caucasians.

The meta-analysis of the recessive genetic model and allele genetic model revealed that the MTHFR C677T polymorphism was significantly associated with the risk of G3-4 nephrotoxicity (*P* < 0.05) (Table 2). While, MTHFR C677T polymorphism wasn’t related to the incidence of G3-4 nephrotoxicity in the recessive genetic model (OR = 1.54, 95%CI: 0.69–3.43) (Table 2).

Similar to G3-4 hepatotoxicity and nephrotoxicity, MTHFR C677T polymorphism was related to the incidence of G3-4 gastrointestinal toxicity and mucositis under the recessive genetic model (G3-4 gastrointestinal toxicity: OR = 1.85, 95%CI: 1.34–2.56; G3-4 mucositis: OR = 1.73, 95%CI: 1.31–2.28) (Table 2). Noteworthy, these results were limited to Asian (Table 2). There was no significant association observed between MTHFR C677T and the incidence of G3-4 hematotoxicity. In the last, Zhou et al. suggested no statistically significant association between MTHFR C677T polymorphism and adverse events overall (*P* > 0.05).

### Correlation between MTHFR A1298C polymorphism and adverse reactions of HD-MTX

Six studies have reported the association between MTHFR A1298C polymorphism and HD-MTX adverse reactions. Because only one study provided specific data on relevant outcome indicators, a descriptive analysis was conducted. Concerning the incidence of hematological toxicity, Windsor et al. found that the MTHFR A1298C polymorphism is significantly correlated with the decrease in hemoglobin (*P* < 0.05). However, Ana Patino-Garcia et al. found no statistically significant association between the two. Park et al., Jabeen et al., and Hattinger et al. found that the MTHFRA 1298C polymorphism has no statistically significant association with G3-4 hepatotoxicity (*P* > 0.05). In addition, Park et al. and Ana Patino-Garcia et al. believed that MTHFR A1298C polymorphism did not have a significant statistical association with G3-4 nephrotoxicity, G3-4 mucositis, gastrointestinal toxicity, and the overall incidence of adverse reactions (*P* > 0.05).

### Correlation between RFC1 G80A polymorphism and HD-MTX adverse reactions

The correlation between RFC1 G80A gene polymorphism and HD-MTX adverse reactions was reported in five studies [16–18, 21]. Meta-analysis was not conducted because there was no overlapped adverse reaction report in five studies. Only Park et al. found that RFC1 G80A polymorphism is significantly associated with G3-4 mucositis in Asian children under the dominant genetic model (AA /GA vs. GG: OR = 0.06). Other studies demonstrated that the RFC1 G80A polymorphism is unrelated to hematotoxicity, hepatotoxicity, nephrotoxicity, and gastrointestinal toxicity.

### Correlation between MDR1 C3435T polymorphism and HD-MTX adverse reactions

Four studies reported the correlation between MDR1 C3435T polymorphism and HD-MTX adverse reactions in the Caucasian population. Similar to RFC1, meta-analysis was not conducted in this genetic polymorphism. Windsor et al. pointed out that the MDR1 C3435T polymorphism is significantly associated with the risk of mucositis under the allele genetic model (C vs. T: OR = 7.5, 95%CI: 1.89–13.12). However, the other three studies suggested that the MDR1 C3435T polymorphism is not significantly associated with HD-MTX adverse reactions (*P* > 0.05).

### Publication bias and sensitivity analysis

We conducted a publication bias analysis of the MTHFR C677T polymorphisms under the recessive genetic model and the incidence of HD-MTX adverse reactions in patients. According to the findings, no substantial publication bias was observed in the effect sizes. Due to the limited number of studies in the meta-analysis, publication bias could not be ruled out completely. The sensitivity analysis results indicated that the study of MTHFR C677T polymorphisms under the recessive genetic model and the incidence of HD-MTX adverse reactions in patients were not trimmed and filled, indicating that the effect size of this meta-analysis study was stable (Table 3).

**Table 3:** Evaluation of publication bias and sensitivity analysis of MTHFR C677T polymorphisms under recessive genetic model

Index	Egger’s regression		Duval and Tweedie’s trim and fill		
	Intercept	*p*	Original effect size	Studies trimmed	Adjusted effect size
G3-4 Hepatotoxicity	-0.936	0.407	1.88 (1.39, 2.56)	0	1.88 (1.39, 2.56)
G3-4 Nephrotoxicity	1.051	0.408	1.72 (1.10, 2.67)	0	1.72 (1.10, 2.67)
G3-4 Mucositis	0.301	0.583	1.73 (1.31, 2.28)	0	1.73 (1.31, 2.28)
G3-4 Hematotoxicity	0.858	0.095	1.67 (0.95, 2.92)	0	1.67 (0.95, 2.92)

## Discussion

Based on the meta-analysis and descriptive analysis of 8 studies, it was found that the MTHFR C677T mutation in osteosarcoma patients may lead to an increased risk of HD-MTX adverse reactions, such as G3-4 hepatotoxicity, G3-4 nephrotoxicity, G3-4 gastrointestinal toxicity, and G3-4 mucositis. This meta-analysis did not find a significant association between MTHFR C677T and G3-4 hematotoxicity in patients with osteosarcoma. Due to the limited number of studies included in the meta-analysis, the subgroup analysis was not thoroughly conducted. However, subgroup analysis based on population stratification revealed that the incidence of HD-MTX adverse reactions induced by MTHFR C677T and HD-MTX might be population-dependent. The MTHFR A1298C polymorphism may be associated with a decreased risk of G3-4 toxicity, including G3-4 hepatotoxicity, G3-4 nephrotoxicity, and G3-4 mucositis, as determined by a descriptive analysis of 5 studies. However, none of these associations were statistically significant. The MTHFR C677T mutation may increase the risk of HD-MTX adverse reactions in osteosarcoma patients. In contrast, the MTHFR A1298C polymorphism may be associated with a decreased risk of adverse reactions (although no statistical significance was observed), which is generally consistent with the findings of a systematic evaluation in patients with hematological malignancies. Based on a descriptive analysis of five studies, this paper found no significant correlation between the RFC1 G80A polymorphism and HD-MTX adverse reactions in osteosarcoma patients which is similar to the findings of a systematic evaluation in patients with hematological malignancies. However, the correlation may be affected by race, age, and the definition and categorization of adverse events. Based on the descriptive analysis of the four studies, only one small sample size study suggested that the MDR1 C3435T mutation might reduce the risk of mucositis. Other outcome indicators, however, did not show any significant correlation. Overall, the correlation between the RFC1 G80A or MDR1 C3435T polymorphism and HD-MTX adverse reactions remains to be determined due to the limited quantity and quality of the included studies.

The mechanism of HD-MTX-induced adverse reactions is largely unknown, akin to most drug reactions. Methotrexate (MTX) binds to plasma proteins at a rate of about 50%, with some metabolized by hepatocytes into polyglutamate MTX (PGMTX), the active form exerting antiproliferative effects [35]. Primarily, MTX is excreted by the kidneys, with a small fraction metabolized in the liver to 7-hydroxy MTX (7-OHMTX) and eliminated via bile [36]. High MTX concentrations often lead to oral mucositis as an initial symptom of toxicity, with tissues like the bone marrow, liver, and kidneys being more prone to HD-MTX-related damage [37]. Accumulation of PGMTX and 7-OHMTX in the liver and kidneys may lead to organ damage. PGMTX, with a stronger affinity for dihydrofolate reductase and enhanced antiproliferative activity, increases the likelihood and severity of adverse reactions [38]. In contrast, 7-OHMTX, less effective than free MTX but poorly water-soluble, tends to accumulate in the kidneys, causing renal injury [39].

## Limitations

The maximum dose of HD-MTX for osteosarcoma is 10 g/m2, which is three times higher than the dose given to patients with malignant hematological tumors. Higher MTX plasma concentrations and extended exposure times result in substantially higher levels of MTX metabolites (such as PGMTX and 7-OHMTX) in patients with osteosarcoma than in patients with malignant hematological tumors. Consequently, the incidence and severity of adverse reactions differ. This study reviewed and integrated the relevant research evidence for osteosarcoma patients in a systematic manner. Nonetheless, the following limitations remain: The sample size and number of relevant original studies were limited, and subgroup analysis was incomplete. In general, the results of the meta-analysis were stable, but publication bias could not be ruled out. To confirm the conclusions of this investigation, it is necessary to further expand the sample size. In addition, the classification and definition of outcome indicators varied between studies. Certain outcome indicators could not be included in the meta-analysis because some studies did not provide specific values for outcome indicators. This study was limited to a descriptive analysis of the adverse reactions caused by HD-MTX in this gene polymorphism. Further multicenter, larger sample size and higher-quality original studies are necessary to evaluate the effect of related gene polymorphisms on HD-MTX adverse reactions in osteosarcoma.

In conclusion, the MTHFR C677T mutation might increase the risk of HD-MTX adverse reactions in osteosarcoma patients (such as G3-4 hepatotoxicity, G3-4 nephrotoxicity, G3-4 gastrointestinal toxicity and G3-4 mucositis). There was no correlation between MTHFR A1298C polymorphism and adverse effects of HD-MTX. The relationship between RFC1 G80A or MDR1 C3435T polymorphisms and HD-MTX adverse reactions was uncertain. In clinical medication, the risk of adverse reactions from HD-MTX should be thoroughly evaluated by combining the detection results of the MTHFR gene with each patient’s clinical situation. When necessary, dose adjustment might maximize the safety of HD-MTX clinical medication and ultimately enhance the clinical outcome for osteosarcoma patients.

### Supplementary Information

**Additional file 1:** **Supplemental table 1.** Baseline characteristics of excluded studies and the reasons of exclusion. **Supplemental table 2.** The detail of NOS quality assessment of included papers.

**Additional file 2:** **Supplemental material 1.** PRISMA 2020 Checklist.

**Additional file 3:** **Supplemental material 2.** Search strategy.

## Data Availability

The datasets used and/or analysed during the current study are available from the corresponding author on reasonable request.

## Abbreviation

HD-MTX: High-Dose Methotrexate
MTHFR: Methylenetetrahydrofolate Reductase
RFC: Reduced Folate Carrier
MDR: Multidrug Resistance Gene
WHO: World Health Organization
CTCAE: Common Terminology Criteria For Adverse Events
NOS: Newcastle-Ottawa Scale
OR: Odds Ratio
CI: Confidence Interval
PGMTX: Polyglutamate MTX
7-OHMTX: 7-Hydroxy MTX
