# Supplementary material for: Correlation between gene polymorphism and adverse reactions of high-dose methotrexate in osteosarcoma patients: a systematic review and meta-analysis
Source: World J Surg Oncol. 2024 Jan 11;22:19. doi: 10.1186/s12957-023-03287-0 (PMC10782754; doi:10.1186/s12957-023-03287-0)
Supplement: Supplementary file 1 — Additional file 1: Supplemental table 1. Baseline characteristics of excluded studies and the reasons of exclusion. Supplemental table 2. The detail of NOS quality assessment of included papers. [file 12957_2023_3287_MOESM1_ESM.docx]

**Supplemental table 1. Baseline characteristics of excluded studies and the reasons of exclusion.**

| **First author, year** | **Country** | **Race** | **Sample size** | **Sex** | | **Gene polymorphism** | **Reasons of exclusion** |
| --- | --- | --- | --- | --- | --- | --- | --- |
|  |  |  |  | **Male** | **Female** |  |  |
| Xie L, 2018 | China | Asian | 59 | 37 | 22 | α, γ | Only the overall survival and the risk of recurrence of the primary disease were reported in this study. |
| Gong Y, 2021 | China | Asian | 80 | 48 | 32 | α, γ | Included pediatric patients were diagnosed with acute lymphoblastic leukaemia and osteosarcoma. Independent analysis of the two different disease was not conducted. |
| Yang F, 2019 | China | Asian | 96 | 53 | 43 | β, δ | Data which reported in the publication was in-sufficient for meta-analysis. And we got no response from author in e-mail request for raw data. |

Note: α = MTHFR A1298C; β = MDR1 C3435T; γ = MTHFR C677T; δ = RFC1 G80A.

**Supplemental table 2. The detail of NOS quality assessment of included papers.**

|  | **Selection** | | | | **Comparability** | | **Outcome** | | | **NOS score** |
| --- | --- | --- | --- | --- | --- | --- | --- | --- | --- | --- |
|  | Representativeness of the exposed cohort | Selection of the non-exposed cohort | Ascertainment of exposure | Demonstration that outcome of interest was not present at the start of the study | Control for the most important factor | Control for any additional factor | Assessment of outcome | Was follow-up long enough for outcomes to occur | Adequacy of follow-up of cohorts |  |
| Hattinger CM, 2016 | 1 | 1 | 1 | 1 | 1 | 0 | 1 | 1 | 1 | 8 |
| Ren HY, 2011 | 1 | 1 | 1 | 1 | 1 | 0 | 1 | 1 | 1 | 8 |
| Windsor RE, 2012 | 1 | 1 | 1 | 0 | 1 | 0 | 1 | 1 | 1 | 7 |
| Jabeen S, 2015 | 1 | 1 | 1 | 0 | 1 | 0 | 1 | 1 | 1 | 7 |
| Park JA, 2016 | 1 | 1 | 1 | 0 | 1 | 0 | 1 | 0 | 1 | 6 |
| Goricar K, 2014 | 1 | 1 | 1 | 0 | 1 | 0 | 1 | 0 | 1 | 6 |
| Xu L, 2018 | 1 | 1 | 1 | 1 | 1 | 0 | 1 | 1 | 1 | 8 |
| Lambrecht L, 2017 | 1 | 1 | 1 | 0 | 1 | 0 | 1 | 0 | 1 | 6 |
| Wei Y, 2022 | 1 | 1 | 1 | 1 | 1 | 1 | 1 | 1 | 1 | 9 |
| Zhou XK, 2013 | 1 | 1 | 1 | 0 | 1 | 0 | 1 | 1 | 1 | 7 |
| Hegyi M, 2017 | 1 | 1 | 1 | 0 | 1 | 0 | 1 | 1 | 1 | 7 |
| Patino-Garcia A, 2009 | 1 | 1 | 1 | 1 | 1 | 1 | 1 | 1 | 1 | 9 |
