# Supplementary material for: Correlation between gene polymorphism and adverse reactions of high-dose methotrexate in osteosarcoma patients: a systematic review and meta-analysis
Source: World J Surg Oncol. 2024 Jan 11;22:19. doi: 10.1186/s12957-023-03287-0 (PMC10782754; doi:10.1186/s12957-023-03287-0)
Supplement: Supplementary file 3 — Additional file 3: Supplemental material 2. Search strategy. [file 12957_2023_3287_MOESM3_ESM.docx]

**Supplemental material 2.Search strategy**

**Pubmed:**

#1

((((((((((((Methotrexate[MeSH Terms]) OR (Amethopterin[Title/Abstract])) OR (Methotrexate, (D)-Isomer[Title/Abstract])) OR (Methotrexate, (DL)-Isomer[Title/Abstract])) OR (Mexate[Title/Abstract])) OR (Methotrexate Sodium[Title/Abstract])) OR (Sodium, Methotrexate[Title/Abstract])) OR (Methotrexate, Sodium Salt[Title/Abstract])) OR (Methotrexate, Disodium Salt[Title/Abstract])) OR (Methotrexate Hydrate[Title/Abstract])) OR (Hydrate, Methotrexate[Title/Abstract])) OR (Methotrexate, Dicesium Salt[Title/Abstract])) OR (Dicesium Salt Methotrexate[Title/Abstract])

#2

(((((((((Osteosarcoma[MeSH Terms]) OR (Osteosarcomas[Title/Abstract])) OR (Osteosarcoma Tumor[Title/Abstract])) OR (Osteosarcoma Tumors[Title/Abstract])) OR (Tumor, Osteosarcoma[Title/Abstract])) OR (Tumors, Osteosarcoma[Title/Abstract])) OR (Sarcoma, Osteogenic[Title/Abstract])) OR (Osteogenic Sarcomas[Title/Abstract])) OR (Sarcomas, Osteogenic[Title/Abstract])) OR (Osteogenic Sarcoma[Title/Abstract])

#3

(((((((((Polymorphism, Genetic[MeSH Terms]) OR (Polymorphisms, Genetic[Title/Abstract])) OR (Genetic Polymorphism[Title/Abstract])) OR (Genetic Polymorphisms[Title/Abstract])) OR (Gene Polymorphism[Title/Abstract])) OR (Gene Polymorphisms[Title/Abstract])) OR (Polymorphism, Gene[Title/Abstract])) OR (Polymorphisms, Gene[Title/Abstract])) OR (Polymorphism (Genetics[Title/Abstract]))) OR (Polymorphisms (Genetics[Title/Abstract]))

#4

(((((("Methylenetetrahydrofolate Reductase (NADPH2)"[MeSH Terms]) OR (Methylenetetrahydrofolate Reductase (NADPH[Title/Abstract]))) OR (Methylene-THF Reductase (NADPH[Title/Abstract]))) OR (Methylenetetrahydrofolate Reductase[Title/Abstract])) OR (5,10-Methylenetetrahydrofolate Reductase (NADPH[Title/Abstract]))) OR (Methylene Tetrahydrofolate Reductase[Title/Abstract])) OR (Tetrahydrofolate Reductase, Methylene[Title/Abstract])

#5

((((((((((Reduced Folate Carrier Protein[MeSH Terms]) OR (SLC19a1 Transporter[Title/Abstract])) OR (Transporter, SLC19a1[Title/Abstract])) OR (Folate Transporter 1[Title/Abstract])) OR (Transporter 1, Folate[Title/Abstract])) OR (SLC19a1 Protein[Title/Abstract])) OR (Protein, SLC19a1[Title/Abstract])) OR (Reduced Folate Carrier[Title/Abstract])) OR (Carrier, Reduced Folate[Title/Abstract])) OR (Folate Carrier, Reduced[Title/Abstract])) OR (Solute Carrier Family 19, Member 1[Title/Abstract])

#6

(((((((((((ATP Binding Cassette Transporter, Subfamily B, Member 1[MeSH Terms]) OR (CD243 Antigen[Title/Abstract])) OR (Antigen, CD243[Title/Abstract])) OR (ATP Binding Cassette Transporter, Sub-Family B, Member 1[Title/Abstract])) OR (PGY-1 Protein[Title/Abstract])) OR (PGY 1 Protein[Title/Abstract])) OR (MDR1 Protein[Title/Abstract])) OR (Multidrug Resistance Protein 1[Title/Abstract])) OR (ATP-Binding Cassette, Sub-Family B, Member 1[Title/Abstract])) OR (ABCB1 Protein[Title/Abstract])) OR (P-Glycoprotein[Title/Abstract])) OR (P Glycoprotein[Title/Abstract])

#7

((MDR1 protein, Filobasidiella neoformans[MeSH Terms]) OR (multidrug resistance protein 1, Filobasidiella neoformans[Title/Abstract])) OR (MDR1 protein, Cryptococcus[Title/Abstract])

#8

#1 AND #2 AND #3

#9

#1 AND #2 AND #4

#10

#1 AND #2 AND #5

#11

#1 AND #2 AND #6

#12

#1 AND #2 AND #7

#13

#2 AND (#3 OR #4 OR #5 OR #6 OR #7)

**The Cochrane library:**

#1

(Methotrexate):MeSH OR (Methotrexate):ti,ab,kw

#2

(Osteosarcoma):MeSH OR (Osteosarcoma):ti,ab,kw

#3

(Polymorphism, Genetic):MeSH OR (Polymorphism, Genetic):ti,ab,kw

#4

(Methylenetetrahydrofolate Reductase (NADPH2)):MeSH OR (Methylenetetrahydrofolate Reductase (NADPH2)):ti,ab,kw

#5

(Reduced Folate Carrier Protein):MeSH OR (Reduced Folate Carrier Protein):ti,ab,kw

#6

(ATP Binding Cassette Transporter, Subfamily B, Member 1):MeSH OR (ATP Binding Cassette Transporter, Subfamily B, Member 1):ti,ab,kw

#7

#1 AND #2 AND #3

#8

#1 AND #2 AND #4

#9

#1 AND #2 AND #5

#10

#1 AND #2 AND #6

#11

#2 AND (#3 OR #4 OR #5 OR #6)

**Web of science:**

#1

(TI=Methotrexate) OR (AB=Methotrexate) OR (KP=Methotrexate)

#2

(TI=Osteosarcoma) OR (AB=Osteosarcoma) OR (KP=Osteosarcoma)

#3

(TI=Polymorphism) OR (AB=Polymorphism) OR (KP=Polymorphism)

#4

(TI=Methylenetetrahydrofolate Reductase) OR (AB=Methylenetetrahydrofolate Reductase) OR (KP=Methylenetetrahydrofolate Reductase)

#5

(TI=Reduced Folate Carrier Protein) OR (AB=Reduced Folate Carrier Protein) OR (KP=Reduced Folate Carrier Protein)

#6

(TI=ATP Binding Cassette Transporter) OR (AB=ATP Binding Cassette Transporter) OR (KP=ATP Binding Cassette Transporter)

#7

#1 AND #2 AND #3

#8

#1 AND #2 AND #4

#9

#1 AND #2 AND #5

#10

#1 AND #2 AND #6

#11

#2 AND (#3 OR #4 OR #5 OR #6)

**China National Knowledge Infrastructure:**

#1

Title, keywords, abstract = Methotrexate

#2

Title, keywords, abstract = Osteosarcoma

#3

Title, keywords, abstract = Polymorphism, Genetic

#4

Title, keywords, abstract = Methylenetetrahydrofolate Reductase

#5

Title, keywords, abstract = Reduced Folate Carrier Protein

#6

Title, keywords, abstract = ATP Binding Cassette Transporter

#7

#1 AND #2 AND #3

#8

#1 AND #2 AND #4

#9

#1 AND #2 AND #5

#10

#1 AND #2 AND #6

#11

#2 AND (#3 OR #4 OR #5 OR #6)

**WANFANG DATA**

#1

Title, keywords, abstract = Methotrexate

#2

Title, keywords, abstract = Osteosarcoma

#3

Title, keywords, abstract = Polymorphism, Genetic

#4

Title, keywords, abstract = Methylenetetrahydrofolate Reductase

#5

Title, keywords, abstract = Reduced Folate Carrier Protein

#6

Title, keywords, abstract = ATP Binding Cassette Transporter

#7

#1 AND #2 AND #3

#8

#1 AND #2 AND #4

#9

#1 AND #2 AND #5

#10

#1 AND #2 AND #6

#11

#2 AND (#3 OR #4 OR #5 OR #6)
